# *Corynebacterium* sp. 2-TD Mediated Toxicity of 2-Tridecanone to *Helicoverpa armigera*

**DOI:** 10.3390/toxins14100698

**Published:** 2022-10-11

**Authors:** Meng Gu, Zhaoxiang Xue, Shenglan Lv, Yuhao Cai, Lei Zhang, Xiwu Gao

**Affiliations:** Department of Entomology, College of Plant Protection, China Agricultural University, Beijing 100193, China

**Keywords:** *Helicoverpa armigera*, *Corynebacterium* sp. 2-TD, 2-tridecanone, metabolism, toxicity

## Abstract

Cotton bollworm (*Helicoverpa armigera*) is a Lepidopteran noctuid pest with a global distribution. It has a wide range of host plants and can harm cotton, tomato, tobacco, and corn, as well as other crops. *H. armigera* larvae damage the flower buds, flowers, and fruits of tomato and cause serious losses to tomato production. Tomato uses the allelochemical 2-tridecanone to defend against this damage. So far, there have been no reports on whether the adaptation of *H. armigera* to 2-tridecanone is related to its symbiotic microorganisms. Our study found that *Corynebacterium* sp. 2-TD, symbiotic bacteria in *H. armigera*, mediates the toxicity of the 2-tridecanone to *H. armigera*. *Corynebacterium* sp. 2-TD, which was identified by 16S rDNA gene sequence analysis, was screened out using a basal salt medium containing a unique carbon source of 2-tridecanone. Then, *Corynebacterium* sp. 2-TD was confirmed to be distributed in the gut of *H. armigera* by quantitative PCR (qPCR) and fluorescence in situ hybridization (FISH). The survival rate of *H. armigera* increased by 38.3% under 2-tridecanone stress after inoculation with *Corynebacterium* sp. 2-TD. The degradation effect of *Corynebacterium* sp. 2-TD on 2-tridecanone was verified by ultra-high-performance liquid chromatography (UPLC). Our study is the first to report the isolation of gut bacteria that degrade 2-tridecanone from the important agricultural pest *H. armigera* and to confirm bacterial involvement in host adaptation to 2-tridecanone, which provides new insights into the adaptive mechanism of agricultural pests to host plants.

## 1. Introduction

*Helicoverpa armigera* is a Lepidopteran noctuid pest with a global distribution. It has a wide range of host plants and can harm cotton, tomato, tobacco, and corn, as well as other crops. *H. armigera* also has high fecundity and strong adaptability, allowing it to cause great harm to crops [1,2]. Although Bt cotton has been widely planted in recent years, cotton bollworm is still very serious on tomato. Cotton bollworm larvae mainly eat the flower buds, flowers, and fruits of tomato, resulting in reduced production of tomato and greatly reduced the commodity of tomato fruit. Plants have evolved defenses to deal with attacks from pests, including *H. armigera*, such as changes in morphology and molecular and biochemical defenses. In the biochemical defense of plants, the production and accumulation of plant secondary allelochemicals is an effective and diverse strategy [3,4]. These plant secondary allelochemicals include nitrogen-containing organics, terpenoids, and phenolic compounds, which defend against pests by disrupting cell membranes, inhibiting ion or nutrient transport, inhibiting signal transduction processes, inhibiting metabolism, and disrupting hormonal regulation of physiological processes [5].

2-tridecanone is a non-alkaloid insecticide isolated from wild tomatoes that has toxic effects on lepidoptera larvae (*Manduca sexta* and *Heliothis zea*) and *Aphis gossypii* [6,7]. 2-tridecanone can protect plants from insect damage and is a good defense against plant secondary substances [8,9]. Even so, *H. armigera* can still feed normally on tomatoes containing 2-tridecanone. Insects are adapted to the host plants not only due to detoxification metabolism or physical defenses of the insect itself but also because of symbiotic bacteria in insects.

The evolutionary pressure of chemical defense substances in plants has caused herbivorous insects to develop corresponding countermeasures. Symbiotic microbes in insects mediate the process when herbivorous insects respond to plant secondary allelochemicals. Insect symbiotic bacteria can provide nutrients for the host to metabolize substances [10], assist the host in feeding [11], and even regulate host reproduction [12]. More importantly, symbiotic bacteria can assist in insect detoxification and metabolism of exogenous toxic substances, such as plant allelochemicals, thus improving the resistance of herbivorous insects to host plants [13].

The symbiotic bacteria in insects can metabolize exogenous toxic substances. For example, diamondback moth (*Plutella xylostella*) gut microbes are rich in *Enterobacter cloacae*, *E. asburiae*, and *Carnobacterium maltaromaticum*, which function directly in the decomposition of plant phenols [14]. Symbionts in cabbage root fly (*Delia radicum*) carry plasmids that contain the saxA gene, which enables them to metabolize the defense metabolite isothiocyanate in crucifers [15]. *Enterococcus faecalis*, *Pseudomonas aeruginosa*, and *Staphylococcus sciuri* in dusky cotton bug (*Oxycarenus laetus*) can survive in high concentrations of gossypol [16]. These findings suggest that these bacteria may have the potential to degrade gossypol. Ben-Yosef et al. [17] found that bacteria in olive flies counteract the inhibitory effect of oleuropein—the principal phenolic glycoside in unripe olives. Pine beetle bacterial symbionts containing genes involved in terpene degradation contribute to the ability of the Mountain pine beetle (*Dendroctonus ponderosae*) to overcome tree defenses by assisting with terpene detoxification [18].

There are many reports on the metabolism of plant secondary allelochemicals by insect symbionts, but no studies on the metabolism of the important plant secondary allelochemical 2-tridecanone by insect symbionts. We speculated that some symbiotic bacteria in *H. armigera* can metabolize 2-tridecanone. We thus screened viable bacteria on a medium containing 2-tridecanone as the sole carbon source. Reinoculation of *H. armigera* with the bacteria showed that the host survival rate to 2-tridecanone was significantly enhanced. In addition, degradation of 2-tridecanone by *Corynebacterium* sp. 2-TD in vitro was detected by ultra-high performance liquid chromatography (UPLC), and *Corynebacterium* sp. 2-TD was confirmed to have a metabolic effect on 2-tridecanone. The above results indicated that *Corynebacterium* sp. 2-TD is crucial for *H. armigera* to deal with the stress of 2-tridecanone.

## 2. Results

### 2.1. Isolation and Identification of 2-Tridecanone Degrading Bacteria

**Figure 1:**
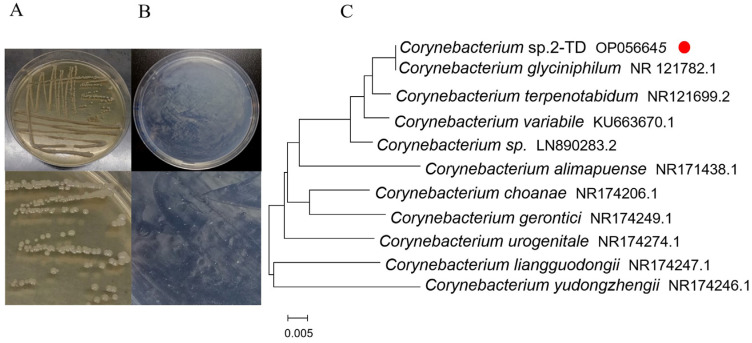
*Corynebacterium* sp. 2-TD isolation and identification. (**A**) The colony characteristics of *Corynebacterium* sp. 2-TD on LB agar plates. (**B**) The colony characteristics of *Corynebacterium* sp. 2-TD on mineral media containing 2-tridecanone. (**C**) Phylogenetic relationships of *Corynebacterium* sp. 2-TD species. The red dot indicates the 2-tridecanone degrading *Corynebacterium* sp. 2-TD strain. The accession numbers of the 16S rDNA sequences for each bacterium are after each species name.

To isolate *Corynebacterium* sp. 2-TD, inoculation was performed on LB agar plates, and the bacteria were identified by 16S rDNA sequencing. After 3 d, *Corynebacterium* sp. 2-TD colonies grown from *H. armigera* isolates were identified by light yellow color, raised center, clean margin, and smooth, wet surface (Figure 1A). *Corynebacterium* sp. 2-TD grew on a mineral medium containing 2-tridecanone (Figure 1B), showing that *Corynebacterium* sp. 2-TD can use 2-tridecanone as the only carbon source and indicating that *Corynebacterium* sp. 2-TD can metabolize 2-tridecanone. 16S rDNA amplification and sequencing yielded a fragment of 1448 bp. Based on a BLAST search against GenBank, the 16S rDNA sequence exhibited 99.72% identity with *Corynebacterium glyciniphilum*. The results of the phylogenetic relationships also show that the bacterium has the highest similarity with *Corynebacterium glyciniphilum* (Figure 1C). The GenBank accession number for this sequence is OP056645.

### 2.2. Localization and Density of Corynebacterium sp. 2-TD in H. armigera

**Figure 2:**
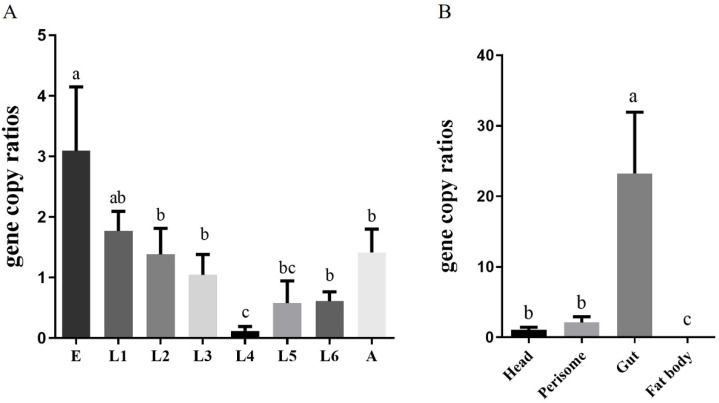
Density of *Corynebacterium* sp. 2-TD in different developmental stages and tissues of *H. armigera*. (**A**) Density of *Corynebacterium* sp. 2-TD in eggs (E), 1st-instar larvae (L1), 2nd-instar larvae (L2), 3rd-instar larvae (L3), 4th-instar larvae (L4), 5th-instar larvae (L5), 6th-instar larvae (L6), and adults (A) of *H. armigera*. (**B**) Density of *Corynebacterium* sp. 2-TD in the head, perisome, gut, and fat body of *H. armigera*. Bars (mean ± SE) labeled with different letters within different stages and tissues are significantly different (*p* < 0.05, independent-sample *t*-tests, *n* = 3).

**Figure 3:**
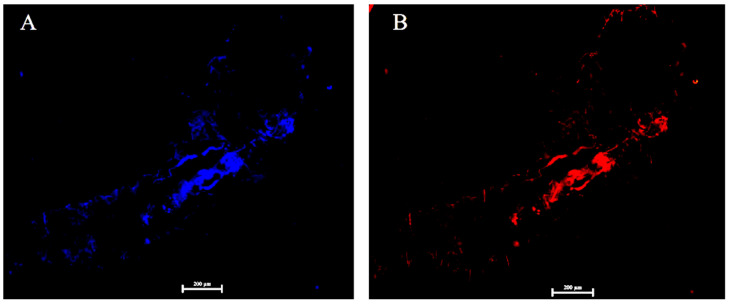
Localization of *Corynebacterium* sp. 2-TD *in H. armigera*. (**A**) Blue signal shows the host insect nucleus. (**B**) Red signal shows *Corynebacterium* sp. 2-TD.

To clarify the density of *Corynebacterium* sp. 2-TD in different developmental stages of *H. armigera*, samples of *H. armigera* from eggs to adults were collected, and their genomes were extracted to detect the density of *Corynebacterium* sp. 2-TD by quantitative PCR (qPCR). The density of *Corynebacterium* sp. 2-TD decreased during development, reaching the lowest density at the fourth instar and then increasing to the adult stage (Figure 2A). We also detected the density of *Corynebacterium* sp. 2-TD in different parts (head, perisome, gut, and fat body) of *H. armigera*, which showed that relative to the head and epidermis, *Corynebacterium* sp. 2-TD was extremely abundant in the intestine, while there was almost no *Corynebacterium* sp. 2-TD in the fat body (Figure 2B). Fluorescence in situ hybridization (FISH) results showed that *Corynebacterium* sp. 2-TD are concentrated in the intestine (Figure 3).

### 2.3. Density of Corynebacterium in H. armigera after 2-Tridecanone Treatment

**Figure 4:**
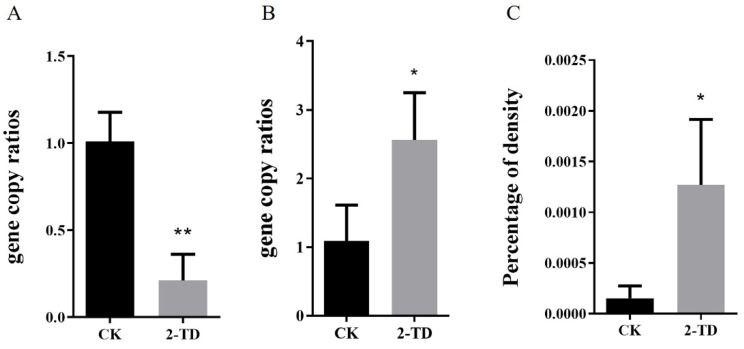
Density of *Corynebacterium* sp. 2-TD in *H. armigera* after short-term 2-tridecanone treatment. (**A**) Density of all bacteria after short-term 2-tridecanone treatment. (**B**) Density of *Corynebacterium* sp. 2-TD after short-term 2-tridecanone treatment. (**C**) Relative density of *Corynebacterium* sp. 2-TD after short-term 2-tridecanone treatment. Asterisks in the figure indicate a significant difference, “*” represents *p* < 0.05, and “**” represents *p* < 0.01.

**Figure 5:**
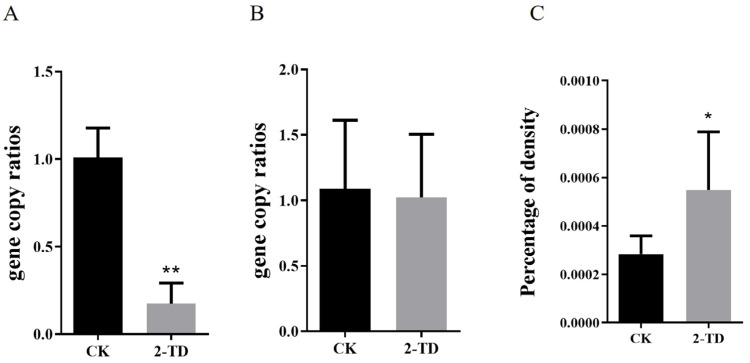
Density of *Corynebacterium* sp. 2-TD in *H. armigera* after long-term 2-tridecanone treatment. (**A**) Density of all bacteria after long-term 2-tridecanone treatment. (**B**) Density of *Corynebacterium* sp. 2-TD after long-term 2-tridecanone treatment. (**C**) Relative density of *Corynebacterium* sp. 2-TD after long-term 2-tridecanone treatment. Asterisks in the figure indicate a significant difference, “*” represents *p* < 0.05, and “**” represents *p* < 0.01.

In order to identify changes in *Corynebacterium* sp. 2-TD density in *H. armigera* after feeding on 2-tridecanone, we first measured the sensitivity of *H. armigera* to 2-tridecanone. The results showed that the lethal concentration 30 (LC_30_) was 2.572 mg/g, LC_50_ was 5.549 mg/g, and LC_90_ was 36.337 mg/g. *H. armigera* treated with 2-tridecanone at the LC_30_ were selected to detect the changes in *Corynebacterium* sp. 2-TD density, and after continuous 5-day short-term treatment, the density of *Corynebacterium* sp. 2-TD increased in *H. armigera* (Figure 4A), the density of total bacteria decreased (Figure 4B), and the percentage of *Corynebacterium* sp. 2-TD in total bacteria increased (Figure 4C). After continuous treatment with an artificial diet containing 2-tridecanone for eight generations, the density of *Corynebacterium* sp. 2-TD did not increase significantly (Figure 5A), but the percentage of *Corynebacterium* sp. 2-TD to total bacteria increased (Figure 5C).

### 2.4. Corynebacterium sp. 2-TD Improve the Survival Rate of H. armigera to 2-Tridecanone

**Figure 6:**
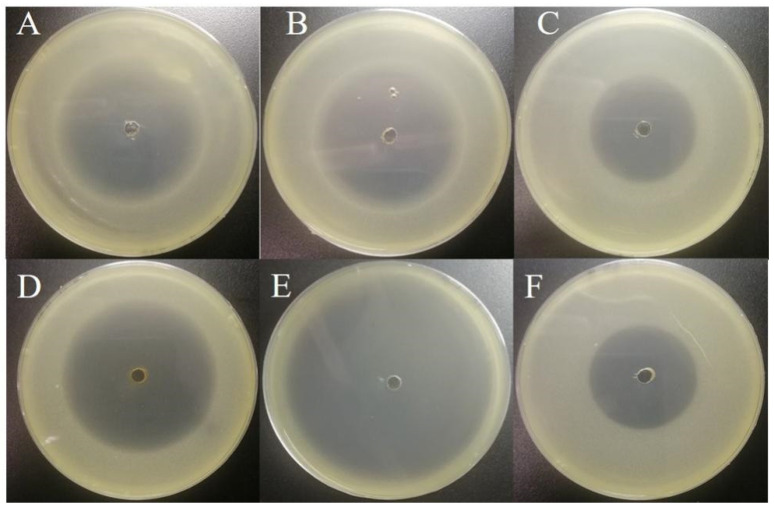
Sensitivity of *Corynebacterium* sp. 2-TD to different antibiotics. (**A**–**F**) Bacteriostatic results of gentamicin, kanamycin, rifampicin, tetracycline, ampicillin, and chloramphenicol against *Corynebacterium* sp. 2-TD, respectively.

**Figure 7:**
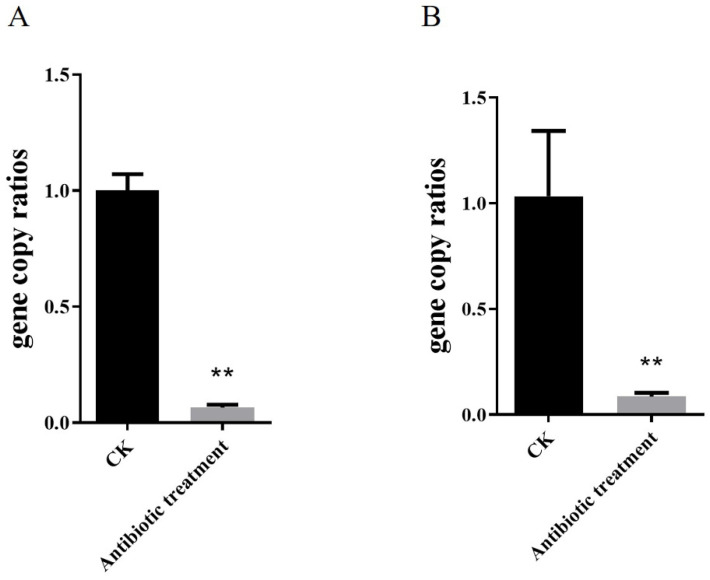
Detection of bacteria elimination effect of antibiotics. (**A**) Density of *Corynebacterium* sp. 2-TD after antibiotic treatment. (**B**) Density of total bacteria after antibiotic treatment. ** represents *p* < 0.01.

**Figure 8:**
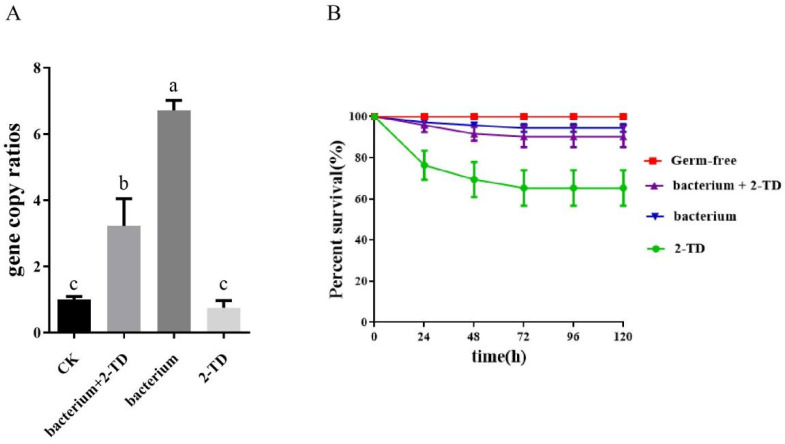
Changes in survival rate of *H. armigera* to 2-tridecanone after inoculation with *Corynebacterium* sp. 2-TD. (**A**) qPCR detection of density after inoculation with *Corynebacterium* sp. 2-TD. (**B**) Survival rate of *H. armigera* to 2-tridecanone after inoculation with *Corynebacterium* sp. 2-TD. Different letters indicate statistically significant differences between groups (*p* < 0.05).

Before inoculation with *Corynebacterium* sp. 2-TD, we eliminated the bacteria in *H. armigera*. First, the susceptibility of *Corynebacterium* sp. 2-TD to different antibiotics was tested. Gentamicin, kanamycin, rifampicin, tetracycline, ampicillin, and chloramphenicol all had inhibitory effects on *Corynebacterium* sp. 2-TD (Figure 6), and the bacteriostatic effect was selected. The more significant antibiotics were gentamicin, kanamycin, tetracycline, and ampicillin, which were mixed into artificial diets fed to newly hatched larvae. After treatment of *H. armigera* with the four antibiotics, the density of *Corynebacterium* sp. 2-TD and total bacteria decreased significantly (Figure 7). After infecting *H. armigera* with inoculum, the bacteria colonized the *H. armigera* effectively (Figure 8A), and the survival rate of *H. armigera* increased by 38.3% under 2-tridecanone stress after inoculation with *Corynebacterium* sp. 2-TD (Figure 8B).

### 2.5. Degradation of 2-Tridecanone by Corynebacterium sp. 2-TD

**Figure 9:**
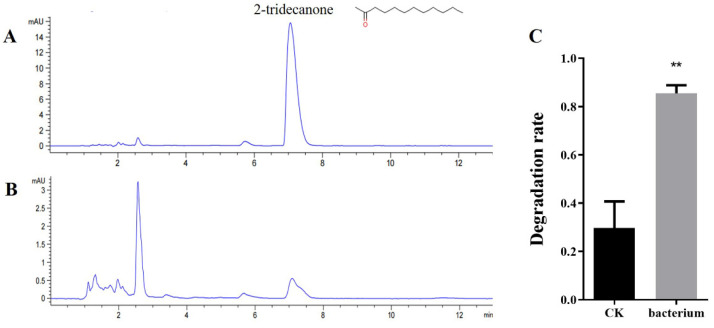
Degradation of 2-tridecanone by *Corynebacterium* sp. 2-TD. (**A**) UPLC identification of 2-tridecanone degradation without *Corynebacterium* sp. 2-TD. (**B**) UPLC identification of 2-tridecanone degradation with *Corynebacterium* sp. 2-TD. (**C**) Degradation rate of 2-tridecanone by *Corynebacterium* sp. 2-TD. ** represents *p* < 0.01.

In order to clarify why *Corynebacterium* sp. 2-TD improves the survival rate of *H. armigera* to 2-tridecanone, we used UPLC to detect the degradation of 2-tridecanone by *Corynebacterium* sp. 2-TD and calculated the degradation rate using the reduction of the parent compound. The test results show that the retention time of 2-tridecone is 7.18 min. After 6 days of reaction, compared with the sample without *Corynebacterium* sp. 2-TD, the 2-tridecanone in the sample system with *Corynebacterium* sp. 2-TD was significantly reduced, which shows that *Corynebacterium* sp. 2-TD had a significant degradation effect on 2-tridecanone (Figure 9).

## 3. Discussion

Reports on the degradation of plant secondary substances by insect gut microbes are common. For example, symbiotic bacteria of *Delia radicum* can degrade isothiocyanates in cruciferous plants [15]. *Candida albicans* in bed bug gut crypts contains the oxalate decarboxylase gene, suggesting that this strain of *Candida* has the potential to degrade oxalate [19]. *Glutamicibacter halophytocola* in the gut of the potato tuber moth (*Phthorimaea operculella*) can degrade α-solanine and α-chaconine in potatoes [20]. This paper is the first to report the isolation of gut bacteria in the important agricultural pest *H. armigera* that degrades 2-tridecanone, the defensive substance of Solanaceae plants. The host plants of *H. armigera* are numerous, but the damage to Solanaceae plants such as tomato is particularly severe. In our study, the bacterium was identified as *Corynebacterium* sp. 2-TD by 16S rDNA sequencing, and the 16S rDNA sequence of the bacterium was 99.72% homologous to *C. glyciniphilum* by BLAST.

*Corynebacterium ulcerus* and *C. diphtheriae* cause respiratory diseases in humans [21]. *Corynebacterium* species are regarded as one of the pathogens causing breast abscesses [22]. In addition, *C. terpenotabidum* Y-11^T^ is a squalene-degrading bacterium [23,24]. *C. glyciniphilum* AJ 3170 was isolated from putrefied bananas in the 1980s [25], and strain AJ 3170 was shown to metabolize dihydroxy acetone, pyruvic acid, D-arabitol, arbutin, salicin, D-glucosamine, and quinic acid as sole carbon sources [26]. *C. glyciniphilum* has recently been found in the intestinal tract of *Oxycarenus laetus*, a cotton pest [16], but the function of the bacterium in host insects is unclear. We isolated *Corynebacterium*, an intestinal bacterium that is highly similar to *C. glyciniphilum*, from *H. armigera* and further confirmed that this bacterium can degrade 2-tridecanone (Figure 1B and Figure 9). The strain thus improves the fitness of the important agricultural pest *H. armigera* to the Solanaceae defense secondary substance 2-tridecanone (Figure 8). In addition, whether strains of other genera in *H. armigera* can also degrade 2-TD is unknown and needs to be further investigated.

Through qPCR quantification and FISH localization, we verified that *Corynebacterium* is mainly distributed in the intestinal tract of *H. armigera* (Figure 3). The functions of insect symbionts are diverse, and the distributions of these symbionts with different roles in the host may also be variable. *Spiroplasma* in *Drosophila melanogaster* accumulate in the ovary, and *Spiroplasma* coopts the yolk transport and uptake machinery to colonize the germ line and ensure efficient vertical transmission [27]. Symbiotic *Rickettsia* of green rice leafhopper (*Nephotettix cincticeps*) is abundantly distributed in the testis. The *Rickettsia* symbiont in *N. cincticeps* efficiently targets and infects the host’s cell nuclei, including sperm head nuclei, and is vertically transmitted to the next host generation not only maternally via ovarian passage but also paternally via intrasperm passage with high fidelity [28]. In the whitefly *Bemisia tabaci*, *Rickettsia* is distributed in the entire body cavity of the insect, as well as in the midgut, salivary gland, ovary, and testis [29]. Most insect symbionts related to detoxification and metabolism are distributed in the intestinal tract and help insects degrade foreign toxic substances. For example, symbiotic yeast in the cigarette beetle, as well as the symbionts *Citrobacter* sp. in *Bactrocera dorsalis* and *Pseudomonas* in coffee berry borer (*Hypothenemus hampei*), are distributed in the host’s gut [30,31,32], which is consistent with our study. We suggest that the degradation site of 2-tridecanone by *Corynebacterium* sp. 2-TD is the intestinal tract. Previous studies have shown that ketone monooxygenase and undecyl acetate esterase in *Pseudomonas* can oxidize 2-tridecanone to undecyl acetate and further catalyze the production of undecanol and acetate by carboxylesterase. Undecanol is oxidized to undecanoic acid, and acetate provides carbon and energy for cellular metabolism through the tricarboxylic acid cycle and the glyoxalate bypass [33,34,35,36]. Whether the degradation of 2-tridecanone by *Corynebacterium* sp. 2-TD occurs through a similar pathway remains to be determined.

The proportion of *Corynebacterium* sp. 2-TD in total bacteria increased significantly after 2-tridecanone treatment (Figure 4 and Figure 5), which confirmed the tolerance of *Corynebacterium* sp. 2-TD to 2-tridecanone. Even the presence of 2-tridecanone favored the survival of *Corynebacterium* sp. 2-TD because the effect of *Corynebacterium* sp. 2-TD was highlighted under the stress of 2-tridecanone. *Corynebacterium* sp. 2-TD with high density improved the response to 2-tridecanone stress in *H. armigera* (Figure 8). Our experimental results show that the density of *Corynebacterium* sp. 2-TD is relatively high in the adult and egg stages of *H. armigera* (Figure 2A), so we speculate that this bacterium may be vertically transmitted in *H. armigera*, but further research is needed. Whether this strain is a gut-specific symbiont of *H. armigera* or is transmitted horizontally, it functions directly in improving the survival rate of *H. armigera* after 2-tridecanone treatment.

Plant secondary defense substances are important factors in insect-plant co-evolution, as they are barriers to insect host transfer and subsequent diversification. By helping insects circumvent these barriers, gut microbial symbiosis may function in generating diversity in herbivorous insects [37]. Insect feeding on plants is related to whether their microbes are good at resisting or detoxifying the plants’ secondary compounds. 2-tridecanone is a natural chemical defense substance of tomato, and this study found that *Corynebacterium* sp. 2-TD functioned directly in the detoxification process of 2-tridecanone. At present, our research is limited to only one host plant secondary allelochemical and one microbe. Further investigations are needed to determine whether there are differences in the microbiota of *H. armigera* from different hosts and whether the microorganisms that play an important role in the defense of different crops are common or specific. 

In addition, symbiotic control related to disruption of insect symbiosis has provided another novel approach to pest control, which can be effectively controlled by manipulation or inhibition of desired culturable symbiotic bacteria for inhibition [20,38]. This technology has been applied to agricultural pests such as olive fruit fly and weevil through antimicrobial peptides [38,39]. *H. armigera* is an important agricultural pest, and its control mainly depends on chemical insecticides. Therefore, the development of sustainable control strategies is highly desirable. Our results provide new insights that pest control relies on disruption of insect symbiosis to control *H. armigera*, but whether the bacteria have a symbiotic relationship with other agricultural pests is unclear. It also remains to be seen whether our results that *Corynebacterium* sp. 2-TD mediated toxicity of 2-tridecanone to *H. armigera* will also have a significant effect in the field, and whether disruption of the bacterium will have adverse effects on the field environment.

## 4. Materials and Methods

### 4.1. Insect Strain and Rearing

The *H. armigera* population used in this experiment is a long-term breeding strain in the laboratory without exposure to any pesticides or phytochemicals. The photoperiod conditions of the whole growth process of *H. armigera* were L:D = 16:8, with temperature conditions of 27 ± 0.5 °C and humidity conditions of 70 ± 10%. The pupae were placed in a cage wrapped with black cloth and covered with clean, sterile white gauze until the adults emerged, mated, and laid eggs. Then, the egg cloth was placed into a clean plastic Ziplock bag until the larvae hatched, and newly hatched larvae were picked into a small box containing artificial feed based on cornmeal and soybean flour in an ultra-clean workbench. When *H. armigera* grew to the third instar stage, the larvae were picked out and placed in a new small box filled with artificial feed to avoid cannibalism until pupation.

### 4.2. Isolation of H. armigera Bacteria

In order to isolate *Corynebacterium* sp. 2-TD, we collected healthy larvae of *H. armigera*, soaked them in 75% ethanol for 1 min, dried them at room temperature for 5 min, then rinsed them with sterile water three times. The dried *H. armigera* larvae were ground into a homogenous bacterial suspension and diluted 5 times at a 10-fold gradient. The obtained bacterial suspension was spread on LB solid medium (yeast extract 5 g/L, sodium chloride 10 g/L, tryptone 10 g/L, and agar 15 g/L) and cultured in a 37 °C incubator for 3–6 days. Morphological characteristics of the colonies were observed, and colonies of different shapes were selected for purification on LB solid medium and purified at least three times.

### 4.3. Screening and Identifying 2-Tridecanone Degrading Bacteria

To verify whether isolated *H. armigera* are resistant to 2-tridecanone, single bacterial colonies that were obtained on LB solid after isolation and purification three times were selected and inoculated on a basic salt medium (1.0 g/L NaCl, 1.0 g/L NH_4_NO_3_, 1.5 g/L K_2_HPO_4_, 0.5 g/L KH_2_PO_4_, 0.1 g MgSO_4_, and agar 15 g/L) [31] containing 100 mg/L 2-tridecanone, and the growth of bacteria was observed every day. Bacteria that could grow on the basal salt medium were recorded and photographed.

Isolated and purified bacteria were transferred to a 2 mL centrifuge tube containing 1 mL of LB liquid medium. Then, the bacteria were identified by 16S rDNA sequencing, and the obtained bacterial 16S rDNA sequences were submitted to the NCBI database for BLAST search to determine the bacterial species. The isolated bacteria were stored in 25% glycerol solution at −80 °C.

### 4.4. Detecting Density of Corynebacterium sp. 2-TD in H. armigera

The relative densities of *Corynebacterium* sp. 2-TD at different developmental stages and in different tissues were characterized. The same samples used for 16S rRNA gene sequencing were assessed for the density of *Corynebacterium* sp. 2-TD using quantitative PCR (qPCR). Specific primers (forward: 5′-GGCTAACTACGTGCCAGCA-3′; reverse: 5′-TACTCAAGTTATGCCCGTATCGC-3′) of the *Corynebacterium* sp. 2-TD 16S rRNA gene were used to determine gene copy numbers. The results were normalized using the *β-actin* gene (forward: 5′-TGTTCGCCGCACTCGCAGTCT-3′; reverse: 5′-GACCGACGATGGACGGGAAGAC-3′) of *H. armigera* to estimate the relative density of the bacteria. The reaction system was SYBR Premix Ex Taq II 10 μL, ddH_2_O 7.4 μL, upstream and downstream primers at 0.8 μL each, and 1 μL template. The quantitative PCR program was 95 °C for 30 s, then 40 cycles of 95 °C for 5 s, 60 °C for 30 s, and finally the dissolution curve. The relative expression formula is F = 2^−∆∆Ct^.

### 4.5. Detecting Distribution of Corynebacterium sp. 2-TD in H. armigera

The distributions of *Corynebacterium* sp. 2-TD in *H. armigera* were visualized by FISH with the following method. Newly hatched larvae were labeled with a Cy3 5’-end fluorescently labeled specific probe (C162:5’-ATCTTTCCAGCATGCGCT-3’) using the *Corynebacterium* sp. 2-TD 16S rRNA gene. *H. armigera* larvae were fixed in Carnot’s solution (ethanol: chloroform: acetic acid = 6:3:1). The immobilized samples were collected and bleached in H_2_O_2_ solution with 6% ethanol, then hybridized overnight in a hybridization buffer containing 40 nM oligonucleotide probes (20 mM Tris-HCl pH 8.0, 0.9 M NaCl, 0.01% SDS, and 35% formamide) at 42 °C. To remove the non-specific binding of probes, samples were washed in wash buffer (20 mM Tris/HCl pH 7.5, 70 mM NaCl, 0.01% SDS, 5 mM EDTA) for 30 min. Finally, the samples were stained for 10 min with DAPI, which contains an anti-fluorescence quencher. Samples were observed under laboratory conditions using an inverted fluorescence microscope.

### 4.6. Antibiotic Treatment and Reinoculation of Corynebacterium sp. 2-TD in H. armigera

In order to detect the susceptibility of *Corynebacterium* sp. 2-TD to antibiotics, we used the method of zone of inhibition. First, a single colony of *Corynebacterium* sp. 2-TD was picked and grown overnight at 37 °C, 200 rpm. Then, 100 μL of the inoculum was mixed with 20 mL LB agar on a plate, and 30 µL of antibiotics was added at a concentration of 1 mg/mL to a 0.3 cm diameter hole in the center of the agar plate. After 48 h of incubation at 37 °C, the size of the inhibition zone was observed. The antibiotics used in this experiment were kanamycin, chloramphenicol, gentamicin, tetracycline, ampicillin, and rifampicin.

Before reinoculating with *Corynebacterium* sp. 2-TD, the newly hatched larvae were fed with four antibiotics, 500 mg/L of ampicillin, kanamycin, gentamicin, and streptomycin, mixed with sterile artificial feed to eliminate the flora in *H. armigera*. Five days later, the *Corynebacterium* sp. 2-TD were used for reinoculation, and the mortality of *H. armigera* was detected after treatment with 2-tridecanone at the lethal concentration 30 (LC_30_). Three replicates were set, with 24 *H. armigera* in each replicate. The entire process was carried out in a sterile environment.

### 4.7. Testing of the 2-Tridecanone Degradation Characteristics of Corynebacterium sp. 2-TD

In vitro metabolism media (MM) were prepared containing four salts: 0.1 g/L NaCl, 0.1 g/L NH_4_NO_3_, 0.15 g/L K_2_HPO_4_, 0.05 g/L KH_2_PO_4_, and 0.01 g/L MgSO_4_. The enrichment medium was prepared by adding 20 mg/mL 2-tridecanone. *Corynebacterium* sp. 2-TD was grown at 37 °C, 200 rpm in the LB medium. After 12,000 rpm centrifugation for 10 min, excess LB medium was rinsed away with sterile water. Then, the inoculum was added to MM (containing 20% acetonitrile); the control was not added to the inoculum. The inoculated media were shaken and cultivated at 37 °C for 6 d. Reactions were stopped by 50% acetonitrile and purified by centrifugation (12,000 rpm, 10 min) and filtration. Cleared samples were analyzed immediately by UPLC. At a rate of 1.0 mL/min, 10 μL of each sample was injected, and 85% acetonitrile and 15% water comprised the mobile phase gradient in gradient mode for the separation. The column was a ZORBAX SB-C18, 4.6 × 150 mm, 5 μm. 2-tridecanone was detected at a wavelength of 300 nm.
